# Post-mortem diagnosis of malaria

**DOI:** 10.1002/nmi2.52

**Published:** 2014-07-15

**Authors:** C Palmiere, K Jaton, A Lobrinus, B Schrag, G Greub

**Affiliations:** 1 University Centre of Legal Medicine, University Hospital Centre and University of LausanneLausanne, Switzerland; 2 Hospital Centre of SionSion, Switzerland; 3 Institute of Microbiology, University Hospital Centre and University of LausanneLausanne, Switzerland; 4 Division of Clinical Pathology, University Hospital Centre and University of GenevaGeneva, Switzerland; 5 Service of Infectious Diseases, University Hospital CentreLausanne, Switzerland

**Keywords:** Chemoprophylaxis, malaria, real-time PCR, sudden death

Dear Editor

Sudden unexpected deaths due to imported malaria occasionally occur in non-endemic areas and can lead to forensic issues 1–4. Post-mortem identification of malaria in non-endemic countries is challenging because pathologists do not often encounter cerebral malaria 5,6 and because the infection is rarely documented ante-mortem. Post-mortem diagnosis of malaria is often only done when there is a suspicion based on anamnesis or available medical information. Unfortunately, medical records are generally incomplete, unreliable or absent when bodies are admitted to the mortuary for medico-legal investigations. Moreover, macroscopic findings at necropsy are often non-specific 7,8.

Here, we report a post-mortem diagnosis of malaria, completely unsuspected clinically. Briefly, a previously healthy 48-year-old Caucasian woman travelled to Ivory Coast with her husband, spending 3 weeks in the southern forest zone. They had taken chloroquine weekly for malaria chemoprophylaxis only before travelling to Africa, due to misunderstanding the medical advice. Three days before returning to Switzerland, she suffered abrupt onset of fever, chills, malaise and muscle aches. Due to flu-like symptoms, she self-medicated with aspirin. Four days after returning from Africa, she was found dead at home. As a result of the unclear death, a medico-legal autopsy was ordered by the prosecutor and performed the same day.

Post-mortem investigations revealed essentially pulmonary oedema, hepatomegaly (2215 g) and splenomegaly (380 g). Toxicology failed to detect common illicit or prescribed drugs. Biochemistry showed increased C-reactive protein (84 mg/L) and procalcitonin (5.08 μg/L). Microbiology failed to detect any underlying bacterial infections.

Given the high suspicion of malaria, urine, vitreous humour, pericardial fluid, blood, liver and brain samples were examined using *Plasmodium falciparum*, *Plasmodium malariae*, *Plasmodium vivax* and *Plasmodium ovale* real-time PCRs 9,10. *Plasmodium falciparum* was confirmed in these samples (Table1). Estimated parasitaemia was 15.2% (760 000 parasites/μL in blood), considering that 50 000 parasites/μL correspond to 1% of parasitaemia 10.

**Table 1:** Quantification by real-time PCR specific for *Plasmodium falciparum* in the different samples investigated

Sample no.	Type of sample	*P. falciparum* real-time PCR	
		DNA copies/mL	Mean value Ct
1	EDTA blood	760 000 000	16.6
2	Liver sample	3 100 000	23.3
3	Pericardial fluid	5200	31.6
4	Urine	200	36.2
5	Vitreous humour	42	38.4
6	Brain sample	29	40.8

Ct, cycle threshold of the specific *P. falciparum* PCR.

Neuropathology showed cerebral oedema (1370 g), grey and white matter with congested vessels containing numerous parasitized erythrocytes with malarial pigment filling the vascular lumen, confirming that the death was due to cerebral malaria. Erythrocytes with malarial pigment were found within dilated vessels in the lungs, myocardium, liver, kidneys and spleen.

In conclusion, malaria is probably an under-recognized aetiology of sudden death in non-endemic countries. In the present case, malaria was only suspected based on the history of recent travel to sub-Saharan Africa, allowing the appropriate investigations to be performed. PCR and Giemsa staining allowed pathologists to confirm the diagnosis and to determine the severity of the disease. This case report underlies the importance of anamnesis in forensic settings. When no information is available and cause of death is not identified, malaria should be considered as a potential aetiology. Moreover, this case underlines the importance of precise advice at the time of anti-malaria chemoprophylaxis prescription, because (1) this patient unfortunately understood that the chloroquine prophylaxis should only be taken before travelling as a preventive measure, and (2) the patient did not ask for medical advice when suffering over several days from a flu-like syndrome, despite her history of travel to a country where malaria is endemic.
